# Performance of convolutional neural networks for the classification of brain tumors using magnetic resonance imaging

**DOI:** 10.1016/j.heliyon.2024.e25468

**Published:** 2024-02-02

**Authors:** Daniel Reyes, Javier Sánchez

**Affiliations:** a Dr. Stetter ITQ S.L.U., Parque Científico Tecnológico, Las Palmas de Gran Canaria, 35017, Spain; b Department of Computer Science, University of Las Palmas de Gran Canaria, Las Palmas de Gran Canaria, 35017, Spain

**Keywords:** Brain tumor classification, Magnetic resonance imaging, Deep learning, Convolutional neural network, Transfer learning, Data augmentation

## Abstract

Brain tumors are a diverse group of neoplasms that are challenging to detect and classify due to their varying characteristics. Deep learning techniques have proven to be effective in tumor classification. However, there is a lack of studies that compare these techniques using a common methodology. This work aims to analyze the performance of convolutional neural networks in the classification of brain tumors. We propose a network consisting of a few convolutional layers, batch normalization, and max-pooling. Then, we explore recent deep architectures, such as VGG, ResNet, EfficientNet, or ConvNeXt. The study relies on two magnetic resonance imaging datasets with over 3000 images of three types of tumors –gliomas, meningiomas, and pituitary tumors–, as well as images without tumors. We determine the optimal hyperparameters of the networks using the training and validation sets. The training and test sets are used to assess the performance of the models from different perspectives, including training from scratch, data augmentation, transfer learning, and fine-tuning. The experiments are performed using the TensorFlow and Keras libraries in Python. We compare the accuracy of the models and analyze their complexity based on the capacity of the networks, their training times, and image throughput. Several networks achieve high accuracy rates on both datasets, with the best model achieving 98.7% accuracy, which is on par with state-of-the-art methods. The average precision for each type of tumor is 94.3% for gliomas, 93.8% for meningiomas, 97.9% for pituitary tumors, and 95.3% for images without tumors. VGG is the largest model with over 171 million parameters, whereas MobileNet and EfficientNetB0 are the smallest ones with 3.2 and 5.9 million parameters, respectively. These two neural networks are also the fastest to train with 23.7 and 25.4 seconds per epoch, respectively. On the other hand, ConvNext is the slowest model with 58.2 seconds per epoch. Our custom model obtained the highest image throughput with 234.37 images per second, followed by MobileNet with 226 images per second. ConvNext obtained the smallest throughput with 97.35 images per second. ResNet, MobileNet, and EfficientNet are the most accurate networks, with MobileNet and EfficientNet demonstrating superior performance in terms of complexity. Most models achieve the best accuracy using transfer learning followed by a fine-tuning step. However, data augmentation does not contribute to increasing the accuracy of the models in general.

## Highlights

• A thorough analysis of convolutional neural networks (CNN) for classifying brain tumors.
• Complexity analysis based on the capacity of the neural networks, training cost, and image throughput.
• Study of the performance of models using data augmentation, transfer learning, and fine-tuning.
• Classification of neural networks performance based on two magnetic resonance imaging (MRI) datasets.

## Graphical abstract

**Figure:**
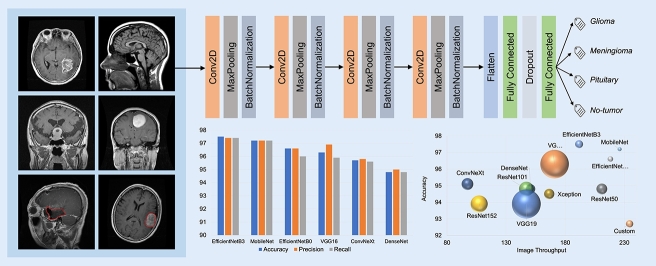

## 1 Introduction

Brain tumors have the lowest survival rate compared to other types of tumors. Unfortunately, less than 7% of patients survive in the most severe cases [1]. The complex nature of these bodies and their implications on the affected area, combined with delayed or incorrect diagnoses, contribute to this outcome. Misdiagnosis often leads to the rapid progression of cancer cells, making direct treatment challenging. In many cases, surgical intervention to remove the tumor becomes unfeasible, leaving chemotherapy as the primary treatment option for these patients.

Early detection of tumors is crucial, and specialists rely on different techniques to evaluate the characteristics of the disease. These techniques include ultrasound scans, computed tomography (CT), magnetic resonance imaging (MRI), and X-ray. Among these techniques, MRI and CT are the most recommended techniques in tumor treatment processes [2]. MRI is the preferred technique because it provides clear images of the internal tissue and is not harmful to the patient.

Over the past twenty years, many research works [3], [4] have introduced novel methods for detecting and classifying tumors through the use of artificial intelligence and computer vision techniques. The primary goal of these methods is to automatically predict pathologies and assist specialists in making informed decisions, ultimately leading to a reduction in costs.

The use of deep neural networks for this task has gained significant attention in recent years. Within a short period of time, promising results have been achieved that allow us to glimpse the development of reliable methods in the future. This progress can be attributed in part to the appearance of various databases with a large number of samples.

Several works in these fields have proposed new architectures especially designed for these types of tumors, whereas many others analyze the benefits of using well-established models. The primary focus is often on optimizing accuracy, while less consideration is given to the complexity and overall performance of the models. Comparing actual approaches is challenging due to the use of various datasets and methodologies that frequently yield contradictory results. This inconsistency raises uncertainties about whether the benefits are derived from the model itself, the dataset configuration, or the methodology employed.

This work aims to present a comprehensive analysis of various convolutional neural networks (CNNs) applied to the classification of brain tumors. To assess the performance of the models, we analyze the training process from different perspectives, such as training from scratch, using data augmentation, and transfer learning. We also use fine-tuning to further increase the accuracy of the models.

The networks analyzed in this study include the *VGG*, *ResNet*, *DenseNet*, *Xception*, *EfficientNet*, *MobileNet*, *DenseNet*, and *ConvNeXt* architectures. We also examine a baseline model composed of a few blocks of convolutional layers, max-pooling, and batch-normalization. Our objective is to evaluate not only the accuracy obtained with these architectures but also their complexity.

This study is based on two recent MRI datasets with more than 3000 magnetic resonance images. The first dataset is available on Figshare and contains 3064 images with three types of tumors: gliomas, meningiomas, and pituitary tumors. The second one is available on Kaggle and contains 3264 images, including an additional label for images without tumors. Both datasets provide sagittal, coronal, and axial views of the brain. The experimental results show that several models achieve state-of-the-art (SOTA) results in both datasets. Among the most accurate networks, we find *EfficentNet*, *MobileNet*, and *ResNet*.

We further investigate the accuracy concerning each type of tumor and find that *meningiomas* are typically more challenging to classify, whereas *pituitary* tumors exhibit the lowest misclassification rate. In terms of complexity, we evaluate the number of parameters, training cost, and image throughput in inference time. This analysis enables us to refine the ranking of our models, establishing a trade-off between accuracy and complexity. Interestingly, several low-capacity models rank high in the classification, while some recent ones demonstrate poor performance.

The contributions of our work can be summarized as follows: a) we perform a thorough analysis of many relevant CNNs for classifying brain tumors using a common methodology; b) we apply the best practices to train the models, such as hyperparameter optimization, data augmentation, transfer learning, and fine-tuning; c) we study the complexity of the models based on the capacity of the neural networks, the training cost, and the image throughput.

Section 3 describes the datasets and neural networks used in this study. Section 4 presents an analysis of the performance of the models, where we compare the accuracy of the most important variants of each network. We estimate the overall accuracy of each model and also examine the results for each type of tumor. Additionally, we evaluate the computational complexity of each network by comparing the training times and the number of parameters of each model. Sections 5 and 6 discuss the results and present ideas for future work.

## 2 Related work

In recent years, there has been significant progress in the classification of brain tumors, mainly due to the application of machine learning techniques, particularly through the use of modern CNNs. Comparative studies have assessed the performance of different CNN architectures, such as Xception, ResNet, Inception, VGG, DenseNet, or MobileNet. These models have demonstrated good accuracy rates for the Figshare [5] and Kaggle [6] datasets.

Nevertheless, these works are not comparable due to the use of different configurations and the lack of details to reproduce the results. In this study, we include most of the models used in these previous works and evaluate their performance on both datasets. In addition, we also consider other networks such as EfficientNet and ConvNeXt, and explore different versions of each family.

### 2.1 Traditional techniques

Before the widespread use of neural networks, traditional techniques for tumor classification involved three main steps: image pre-processing, feature extraction, and image classification, each using different techniques. Typical pre-processing techniques included noise removal, contrast enhancement, edge detection [7], or median filtering [8].

Many feature extraction methods were usually combined, such as the image histogram with the gray level co-occurrence matrix (GLCM) in [9] and multivariate statistical analysis in [3], or the segmentation of the image with GLCM in [8]. The work in [10] was focused on magnetic resonance spectroscopy (MRS) instead of MRI, with features based on frequency alignment, phase correction, and filtering of the dominating residual water peak with the Fast Fourier Transform (FFT). Wavelet-based features, on the other hand, were also used in [11]. Another example is the work presented in [12], where the authors combined features based on the tumor shape, image intensity statistics, and rotation-invariant Gabor texture features.

Several works compared the performance of different machine learning algorithms, such as the comparison of Random Forest, k-Nearest Neighbor (KNN), AdaBoost, and RusBoost in [11]. Linear discriminant analysis (LDA) was used in [4] and in [10] for classifying MRS. Support vector machines (SVM) were probably the most used techniques for classification, such as in [3], [4], [10], or [7]. The work presented in [13] compared SVMs with fuzzy neural networks and found SVM to provide superior performance. The method presented in [9], on the other hand, showed that bag-of-words (BoW) provided better results than other classification techniques.

This latter method introduced the Figshare dataset [14], which has been used in many subsequent works. BraTS [15] is another common dataset that has been used in some previous works [7], [8], [11], although it is oriented to binary classification. These traditional techniques have several limitations. On the one hand, it is difficult to design features that correctly represent the space of the input data and, thus, they usually fail in unexpected situations. On the other hand, combining different techniques usually leads to complex pipelines that do not adapt well to small variations in the data. This complexity increases with the number of techniques, where it is difficult to understand how the parameters of each method affect the final results. For these reasons, traditional strategies have been superseded by neural networks, which achieve higher accuracy and are end-to-end trainable.

### 2.2 Hybrid methods

Many techniques combine neural networks with traditional methods in two typical configurations: using the neural network as a backbone for feature extraction or as the final classifier with features generated by traditional techniques. The latter approach allows for a smaller training set and the design of models with lower capacity, as they rely on more discriminative features. One of the first hybrid methods [16] was based on non-linear least squares features and a probabilistic neural network.

Different neural networks have been used for feature extraction, like a simple convolutional network in [17], GoogLeNet in [18] or DenseNet201 in [19]. The preferred technique for classification was SVM, although several works have compared SVMs with other classifiers, like KNN in [18], XGBoost and Extreme Learning Machine (ELM) in [17], or a combination of a genetic algorithm in [19]. The work presented in [20] concatenated the output of three CNNs and analyzed nine different classifiers, obtaining the best results with an SVM.

Some of these works attained high accuracy with the Figshare dataset [17], [18] and the Kaggle dataset [20], although it is possible to obtain similar results using a single neural network.

Several works combined traditional techniques for feature extraction and neural networks for classification, such as in [21], with features based on the discrete wavelet transform (DWT), Fuzzy C-means clustering, and principal component analysis (PCA), or in [22], with DWT and Gabor filters. In these cases, a multilayer perceptron was used to process the features. The main benefit of this approach is that it is possible to train the model with fewer images, like the 66 MR images used in [21].

The work presented in [23] used the neural network in the middle of the pipeline: the images were segmented with Fuzzy C-Means Clustering and deformable snakes; then, the features were extracted using a CNN with ensemble classification; finally, the classification was carried out with an SVM. A similar work [24] relied on K-Means clustering, edge-based texture histogram equalization, and the discrete cosine transform (DCT) to extract features, VGG16 and VGG19 to process the features, and ELM for carrying out the final classification.

Although some of these methods achieved high classification ratios, in many cases their performance is not as good as recent CNNs. Hybrid methods share limitations with traditional techniques since they are difficult to configure and train. Furthermore, the neural networks must be trained, and their hyperparameters have to be tuned, whenever the settings of other techniques in the pipeline are modified.

### 2.3 Neural networks

The use of CNNs has been studied in various works [25], [26], obtaining good performance for the Figshare dataset. In the work presented in [27], for instance, the authors obtained the best performance using a basic CNN rather than a fully connected neural network or Random Forest. In our study, we analyze a similar network in order to verify these results.

Several works have also analyzed the performance of standard architectures, like GoogLeNet [28] and AlexNet [29] in [30], which demonstrated the superiority of GoogLeNet using transfer learning. Capsule networks have also been used in several works [31], [32], [33], although their accuracy on the Figshare dataset is far from the best models.

ResNet50 and EfficientNetB0 have shown to provide good accuracy in [34] and [35], respectively. In the first work, the authors obtained the best results without data augmentation, which aligns with our results. In the case of EfficientNet, we obtained better results with several models of this family using fine-tuning. A recent work [36] obtained high accuracy using various metaheuristic methods to optimize the hyperparameters of a ResNet50 model and the method proposed in [37] relies on Particle Swarm Optimization (PSO) to search for the optimal hyperparameters of a simple CNN architecture. The authors obtained good accuracy for the binary classification of brain tumors.

More recently, vision transformers (ViT) [38] have been analyzed in [39], obtaining high accuracy, although with an ensemble model and larger images. The main drawback of the transformer architecture is that it has many more parameters, requiring higher computational costs for training. In this work, several models performed better with fewer parameters, as detailed in Sect. 4.4. Generative adversarial networks (GANs) have been used in [40], where a CNN was pre-trained to learn the structure of MR images and then fine-tuned to classify the tumors.

There are also various works that combine different architectures, like DenseNet and LSTM in [41], the first one to extract features from each slice and the second one to integrate the features of the 3D MRI volume. A similar work combines the Xception network with two attention modules [42], the first one to extract spatial features, and the second one, based on a ConvLSTM network, to learn dependencies of the spatial features.

Inception ResNet and Xception were combined in [43] using a mechanism to extract the most interesting features of each network. The method proposed in [44] uses a multiscale approach with three scales of different resolutions, each composed of two convolutional layers and max-pooling. The outputs were concatenated to feed another convolutional layer with max-pooling. Finally, the result was processed through densely connected layers.

More recently, an ensemble of three models was proposed in [45], based on VGG16, Inception, and Xception, obtaining a high accuracy with the Kaggle dataset. The authors also studied three different vision transformers, but they did not obtain satisfactory results. Another work [46] proposed an ensemble method based on five CNNs using various datasets.

Although the accuracy of these methods is typically above 90%, it is not easy to compare their results because they use different datasets and configurations. In this article, we use a common framework to compare the neural networks. The comparison in Sect. 4.4 shows that similar, or even higher, accuracy can be obtained with simpler architectures.

A comparison between some of these works is detailed in Table 9, where we compare our results with those of the SOTA methods. More information about the features and limitations of many of these works can be consulted in [37] or [45].

## 3 Materials and methods

The World Health Organization (WHO) has reported [47] over 120 types of tumors in the central nervous system. Tumors are abnormal masses of tissue that cause swelling and distention in the affected area. They can be classified as benign (noncancerous) or malignant (cancerous). Malignant tumors can spread to other parts of the body and pose a risk to the patient's life.

There are three main types of brain tumors: *glioma*, which is formed from glial cells or cells of the viscous support that surrounds nerve cells; *meningioma*, which is the most common brain tumor and arises from the meninges; and *pituitary* tumors, which is found in the pituitary gland and is responsible for producing hormones related to growth and other glands.

This section provides an overview of the datasets used in our study and how the images are organized into the training, validation, and test sets. Then, we explain the architectural details of the neural networks used in this work. Finally, the experimental setup deals with the runtime environment, the configuration of the training process, the optimization of hyperparameters, and the metrics used to evaluate the methods.

### 3.1 Datasets

#### 3.1.1 Figshare brain MRI dataset

**Figure 1:**
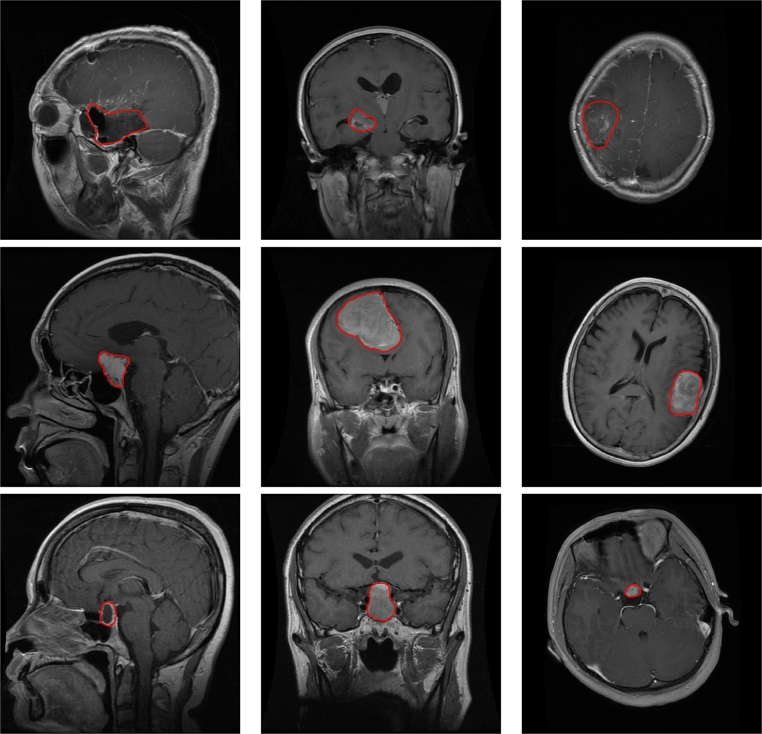
Images from the Figshare dataset: the first row shows three images of *gliomas* from a sagittal plane on the left, a coronal plane in the center, and an axial plane on the right; the second row shows three images of *meningiomas* from sagittal, coronal and axial planes; and the third row shows images of *pituitary* tumors from the same views.

In this work, we use the Figshare dataset [9], which is composed of 3064 T1-Weighted images with the three types of tumors. Each file contains a data structure with the MR image, the tumor type, the coordinates of its contour, and a mask with the segmentation of the tumor region. The dataset contains 1426 images of gliomas, 708 images of meningiomas, and 930 images of pituitary tumors. The data is stored in Matlab files, so we previously converted them to PNG format on disk. Fig. 1 shows several slices from different views.

The images of the dataset were randomly organized in three sets, with 80% for training and 10% for validation and testing. Table 1 shows an example of a random split of the dataset. The training set was used for learning the parameters of the models and the validation set was used for fine-tuning the hyperparameters. The test set was used for assessing the performance of the models in the experiments.

**Table 1:** Split of the Figshare dataset into the training, validation, and test sets. The training set contains 80% of the images, and the validation and test sets contain 10% each. We show the number of images corresponding to each type of tumor for a given random split.

Tumor type	Training	Validation	Test	Total
Glioma	1142	141	143	1426
Meningioma	572	65	71	708
Pituitary Tumor	737	100	93	930

Total	2451	306	307	3064

#### 3.1.2 Brain tumor classification (MRI) dataset

The second dataset [48], from Kaggle,^1^ contains 3264 slices, with 926 gliomas, 937 meningiomas, 901 pituitary tumors, and 500 no-tumors. This dataset includes images from other sources, especially from the Figshare dataset, where we found more than 2260 images of coincidence between the two datasets.

Since many images had arbitrary sizes, we scaled the images to 512×512 in a pre-processing step: in the first step, we selected the largest dimension of the image and scaled it to 512 pixels; then, we centered the image and introduced black regions on both sides to complete the other dimension to 512 pixels.

**Figure 2:**
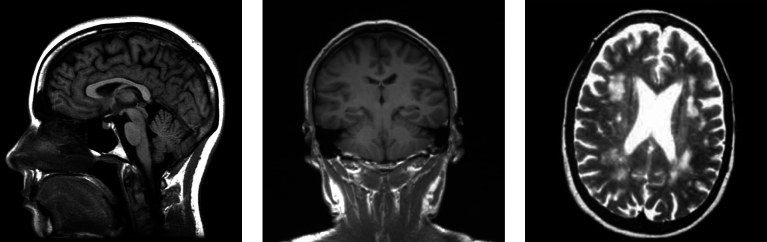
Images from the Kaggle dataset: three images of the *no tumor* class. The first image on the left corresponds to a sagittal plane, the center image to a coronal plane, and the right image to an axial plane. The other classes in this dataset are similar to the Figshare dataset shown in Fig. 1.

**Figure 3:**
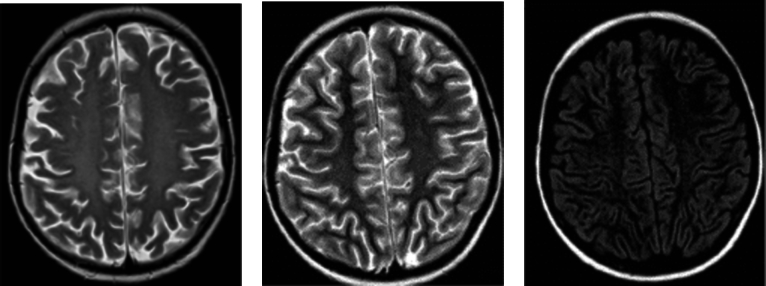
Types of MR images in the Kaggle dataset: a T1-Weighted type image is shown on the left, a T2-Weighted type image in the center, and a Flair-type image on the right.

This database comprises MR images with various types of resonances from axial, sagittal, and coronal planes; see Fig. 2. Additionally, it encompasses three MRI physics variations: T1-Weighted, T2-Weighted, and Fluid Attenuated Inversion Recovery (Flair); see Fig. 3. These resonances are derived from different exposure times.

T1-Weighted images employ the shortest duration of time, predominantly displaying tissue information. Consequently, they provide less information about anomalies compared to the other types of resonances. T2-Weighted images utilize longer exposure times, allowing for clearer extraction of properties and the visualization of cerebrospinal fluid (CSF) along with tissue details. Flair, on the other hand, employs longer exposure times, which darkens the CSF and highlights anomalies.

An example of the split of the database into the training, validation, and test sets is given in Table 2, with 80% for training, 10% for validation, and 10% for testing.

**Table 2:** Split of the Kaggle dataset into the training, validation, and test sets. The training set contains 80% of the images, and the validation and test sets contain 10% each. We show the number of images corresponding to each type of tumor for a given random split.

Tumor type	Training	Validation	Test	Total
Glioma	752	81	93	926
Meningioma	740	103	94	937
No tumor	398	52	50	500
Pituitary tumor	721	90	90	901

Total	2611	326	327	3264

### 3.2 Neural networks

In this study, we consider the following neural networks: *VGG*, *ResNet*, *Xception*, *DenseNet*, *MobileNet*, *EfficientNet*, and *ConvNext*. We explore different models within each architecture.

#### 3.2.1 Custom network

**Figure 4:**
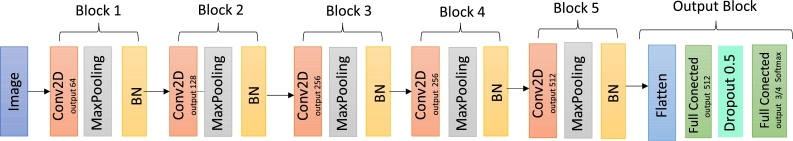
Architecture of the *Custom* neural network: This network contains five blocks of convolutional, max-pooling, and batch-normalization layers, and two fully connected layers at the top. The output is a softmax layer with three neurons for the Figshare dataset and four neurons for Kaggle.

We analyze a custom-designed CNN, called *Custom*, which is composed of five blocks of convolutional, max-pooling (MP), and batch-normalization (BN) layers, as shown in Fig. 4. The first convolutional layer has an output filter of 64, a 3×3 kernel size, a default stride of 1×1, same-type padding, and rectified linear unit (ReLU) activation. The filters of the rest of the convolutional layers are 128, 256, 256, and 512, respectively.

We tried several configurations using a range of filters between 64 and 1024 in each layer but we found this setting to be appropriate, although some other configurations provided similar results. We also tested with a different number of layers but found that five convolutional blocks were typically more precise. We also swapped the order of the layers, putting BN before the other layers, and also before the ReLU activation function, but we did not find significant differences.

Each convolutional layer is followed by a 2D max-pooling subsampling layer with a kernel of size 2×2. The output of the last convolutional layer is flattened and passed through a dense layer of 512 neurons, a dropout of 50%, and an output of three or four neurons with softmax activation. This architecture is somehow similar to AlexNet [29], but we use smaller convolutional kernels, BN, and dropout. The purpose of this network is to establish a baseline model so we can understand the benefits of using more complex architectures.

#### 3.2.2 VGG

The *VGG* architecture [49] is one of the first neural networks with a large number of layers. It generalizes the use of small filter kernels of size 3×3 and combines five blocks of several convolutional layers with MP. These blocks reduce the size of the input data at the same time that they double the number of filters, from 64 to 512. At the top of the model, there are three fully connected layers of 4096, 4096, and 1000 neurons, with two dropout layers, and softmax. In this work, we use the VGG16 and VGG19 models, composed of 16 and 19 layers, respectively.

#### 3.2.3 ResNet

Several configurations of *ResNet* [50] are also studied in this work, characterized by having many convolutional layers, small filter kernels, and skip connections. We analyze networks with 50, 101, and 152 layers.

This model is composed of a first convolutional layer with 64 filters, a kernel size of 7×7, and MP. Similar to VGG, the rest of the convolutions reduce the size of the input and double the number of filters with a constant kernel size of 3×3. The main structure follows two paths, one for the skip connections, represented as identity mappings, and another for residual functions composed of two blocks of convolutional layers, BN, and ReLU activation functions. The architecture is composed of four blocks of convolutional layers. The last layers combine average pooling with a fully connected layer and softmax. The different models of this network increase the number of convolutions in the two internal blocks.

Additionally, we also studied the second version of *ResNet* proposed in [51]. The main difference with respect to the first version is that it changes the residual functions with full pre-activation residual functions composed of two blocks of BN, ReLU, and convolutional layers. In the experiments, we report the results of this version, as it consistently provides better results than the previous one.

#### 3.2.4 Xception

The *Xception* network [52] relies on depthwise separable convolutions, BN, and residual connections. It is inspired by the *VGG16* model, with kernels of size 3×3, by the Inception architecture [28], with separate convolutions for the channel and spatial dimensions, and also by the residual connections of *ResNet*.

This model is composed of 36 convolutional layers in fourteen different modules with linear residual connections. It uses global average pooling after the convolutional backbone, and dropout of 50% before the last fully connected layer. The separate convolutions contribute to reducing the number of parameters and speeding up the training process, obtaining better results than Inception and ResNet in the ImageNet dataset.

#### 3.2.5 DenseNet

*DenseNet*[53] connects all the layers to each other so that the input of every layer is composed of the outputs of all previous layers. Input features are concatenated instead of added as in the ResNet family. The network size is reduced by using a small number of filters per layer. This network alleviates the vanishing-gradient problem, strengthens feature propagation and reuse, and reduces the number of parameters. It usually provides good results for small datasets.

It relies on bottleneck layers with BN, ReLU, and convolutions with kernels of size 1×1 and 3×3. It also introduces transition layers between blocks with BN, a convolutional layer of 1×1, and average pooling. In the original work, the authors studied different configurations with 121, 169, and 201 layers. In this work, we use the model with 201 layers.

#### 3.2.6 MobileNet

*MobileNet*[54] was designed for devices with memory constraints and low computing power. Similar to the *Xception* network, it is based on depthwise separable convolutions and also relies on two hyperparameters for adapting the size of the network and reducing the computational cost. These hyperparameters establish a trade-off between latency and accuracy.

The first version of this family has twenty-eight layers. In the second version [55], the basic building block is a bottleneck depth-separable convolution with residuals and is composed of seventeen residual bottleneck layers and three standard convolutional layers. It uses BN after each layer.

The third version [56] employs a platform-aware neural architecture approach to find the global network structures. It defines a multi-objective optimization problem, based on the accuracy and latency, to obtain a base architecture. Then it uses the NetAdapt algorithm [57] to adapt the size of the network. The experiments on ImageNet show that this version is slightly more accurate than the previous ones while reducing latency. In the experiments, we show the results of the first version, since it provided the best results for our datasets.

#### 3.2.7 EfficientNet

*EfficientNet*[58] defines a strategy to scale the size of the network in order to optimize the accuracy and the number of FLOPS. It performs a compound scaling of the depth and width of the network and the resolution of the input images to maintain a trade-off between accuracy and FLOPS. The first version of this family is composed of eight different models, from B0 to B7, with an increasing number of layers and parameters.

This network relies on the MnasNet architecture [59], which uses inverted bottlenecks [55], and squeeze-and-excitation optimization [60].

In our study, we analyze the second version of this architecture [61], which introduces several improvements over the previous one. In particular, it introduces *progressive learning*, where the size of the images and strength of regularization is gradually increased during training; it replaces depthwise convolutions in the first layers to increase the training speed; and it gradually scales the networks starting from the last stages.

We report the results of the second version, in particular for the B0 and B3 models, since its accuracy is consistently better than the first one. The number of parameters is typically lower than other neural networks with similar performance.

#### 3.2.8 ConvNeXt

*ConvNeXt*[62] is a recent CNN that introduces several improvements taken from vision transformers. The design of this network starts from the ResNet model and borrows some ideas from the vision transformer.

It uses the AdamW [63] optimizer for training the neural network, with many epochs, and up-to-date data augmentation and regularization strategies. It adopts depthwise convolutions and an inverted bottleneck design. Additionally, it increases the kernel size to 7×7 and substitutes BN with Layer Normalization.

They take a few more design decisions, like replacing ReLu with GELU activation functions, increasing the width of the network, and reducing the number of normalization layers and activation functions.

The authors of this work demonstrate that these improvements allow CNNs to outperform the Swin Transformer [64] and DeiT [65] in several tasks, such as image detection, classification, or segmentation.

Our study involves selecting a broad range of CNNs, including multiple models from the same families but with different capacities. We start with a small neural network that has five convolutional layers and then include deeper neural networks with significant architectural differences. For instance, VGG increases the number of layers to 16 and 19 and uses of 3×3 kernels extensively. ResNet introduces residual connections and considerably increases the number of layers to 50, 101, and 152. Xception improves the performance of layers with depthwise separable convolutions. DenseNet redefines the concept of skip connections from ResNet by concatenating the features of previous layers. MobileNet, on the other hand, relies on bottleneck depth-separable convolutions and optimizes its size based on a balance between accuracy and latency. EfficientNet optimizes the size of the network based on both accuracy and the number of FLOPS. Finally, ConvNeXt is founded on ResNet and adopts several design decisions of vision transformers, such as using layer normalization, GELU activation functions, and some ideas to improve the training process.

### 3.3 Experimental setup and metrics

For the implementation of the models, we used the TensorFlow and Keras libraries. Part of the evaluation was also carried out with the Scikit-learn library. The code is available on GitHub^2^

The experiments were conducted on Google Colab and on a desktop computer with an Intel Core i9-10940X CPU @3.30GHz processor with 32GB RAM, an NVIDIA Geforce RTX 2060 GPU with 8GB RAM, and another NVIDIA Geforce RTX 3060 GPU with 12GB RAM, under the Windows 10 Operating System.

#### 3.3.1 Data preprocessing

During the experiments, the images of the datasets were previously scaled-down and augmented to three color channels, resulting in images of size 256×256×3. Then, the pixel values were normalized between -1 and 1. The transformations used for data augmentation were horizontal flipping, in-plane rotations, and zooming. In the last two cases, the empty regions were filled with a constant value of -1, which corresponds to the background color of the MRI slices. The range of random rotations was between -10% and 10% of 2*π*, and the range of zoom transformations was between -20% and 20%.

#### 3.3.2 Hyperparameter optimization

During our research, we conducted an optimization process to find the best hyperparameters for each model, using the validation set. For our custom convolutional network, we experimented with various configurations of convolutional layers, batch-normalization, and max-pooling, varying the order and number of layers, as well as the number of features. We also tested several fully connected layers and dropout on top of the CNN and found that five convolutional blocks with two fully connected layers generated the best results. Some configurations provided similar results, especially for different numbers of features in the last convolutional blocks, so we chose one of the models with the least number of parameters. The optimization process led us to the neural network depicted in Fig. 4.

We also searched for the best hyperparameters of the other CNNs. In these cases, we retained the backbone of the networks and introduced several fully connected layers and dropout on top of the models. We conducted extensive experiments, changing the number of fully connected layers, the number of neurons in each layer, and the dropout rates from 25% to 70%. We found that two fully connected layers produced good results in many cases, but it also led to overfitting. To address this issue, we increased dropout and weight decay, but it was challenging to find a configuration that behaved well for all the models. As a result, we tested one fully connected layer, which is the default configuration for several standard networks, and found that it produced satisfactory results for many models. This setting allowed us to overcome any over- and underfitting problems, as outlined in the results. For simplicity, we used this setting for all the models during the experimental results.

Additionally, we optimized the parameters of the networks with the Adam and RMSProp optimizers and did not find significant differences. We found that Adam was more stable and slightly more accurate than RMSProp in several cases using default parameters.

#### 3.3.3 Configuration of the training process

The experimental results section below is organized into four principal sections: the first one compares the accuracy of the models using the two datasets; the second one studies the performance of the models with respect to each type of tumor; the third one analyzes the trade-off between accuracy and model complexity, studying the memory requirements, the training time, and the throughput of each model; finally, the fourth section compares our results with SOTA methods based on the datasets used in each work.

In the first part, we compare two main scenarios: training the models from scratch and using transfer learning, both with and without data augmentation. Then, we explore the benefits of using fine-tuning after transfer learning.

The training and validation sets were organized in batches of size 32, randomly sampled from the datasets. The number of epochs was thirty when the models were trained from scratch, or using transfer learning, and forty-five when using data augmentation.

We used the Adam optimizer with a learning rate of 0.001, $\beta_{1}=0.9$, and $\beta_{2}=0.999$. The learning rate of the Adam optimizer for fine-tuning was reduced to $10^{-4}$.

The weights used for transfer learning corresponded to the weights obtained with the Imagenet dataset [66]. During the training process, the weights of all layers were frozen except the top of the neural network, which was replaced by a fully connected layer. In particular, this was composed of a flatten layer to accommodate the output of the CNN, a dropout layer with a rate of 50%, and a softmax layer with the corresponding number of classes (three for Figshare and four for Kaggle).

In the fine-tuning step, some layers of the CNN backbone were unfrozen to let their weights adapt to the training data. The models were trained for fifteen more epochs using the training and validation sets together. In some cases, all the layers were unfrozen or just the last fifteen layers, depending on the improvement in accuracy, as commented in the experimental results. The experiments were executed five times with different random seeds and the results are reported in Sect. 4 for the highest accuracy.

#### 3.3.4 Loss function and metrics

We used one-hot encodings for the labels, with glioma represented as (1,0,0), meningioma as (0,1,0), and pituitary tumors as (0,0,1). The loss function used during the optimization process was categorical cross-entropy, which is given by:

(1) $$loss(y,\overset{ˆ}{p})=-\frac{1}{N}\sum_{n=1}^{N}\sum_{i=1}^{C}(y_{i}^{n}\log{\overset{ˆ}{p}}_{i}^{n})$$

with *N* the number of images in the batch, *C* the number of labels (three for Figshare and four for Kaggle), $y_{i}^{n}$ the probability of label *i* for image *n*, and ${\overset{ˆ}{p}}_{i}^{n}$ the prediction of label *i* for image *n*.

We used the accuracy metric for studying the performance of the training, validation, and test sets. This measure represents the percentage of images that have been correctly classified and is calculated as:

(2) $$accuracy=\frac{TP+TN}{TP+FP+TN+FN},$$

with *TP* the number of true positive classifications, *TN* the true negatives, *FP* the false positives, and *FN* the false negatives.

We used the precision and recall metrics to study the performance of the models with respect to each type of tumor. The precision measures the rate of positive predictions that are correctly classified and is calculated as:

(3) $$precision=\frac{TP}{TP+FP},$$

and the recall measures the rate of positive predictions that are correctly classified with regard to the true positive labels and is given by:

(4) $$recall=\frac{TP}{TP+FN}.$$

**Figure 5:**
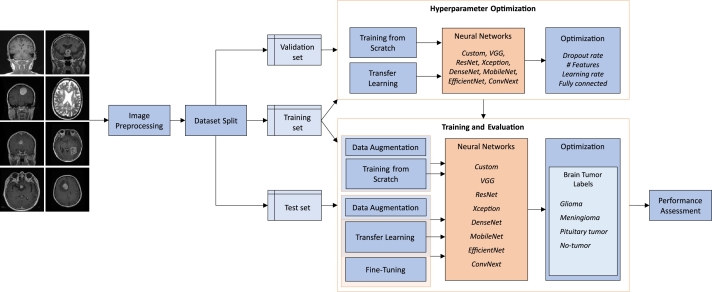
Diagram with the main steps of the methodology. On the left, we start with the images of the dataset that are resized and normalized after reading from the disk. The dataset is then split into the training, validation, and test sets. The training and validation sets are used for finding the optimal hyperparameters, as depicted in the top-right corner of the diagram. The training and test sets are used for training and evaluating the models based on the selected hyperparameters. The output of the models with the test set is used in the performance assessment process. Each block shows the combinations of data augmentation, training from scratch, transfer learning, and fine-tuning used in each setting.

We use the test set of each dataset for calculating these metrics in the experimental results. Fig. 5 depicts the methodology that we have followed in this study. The images of the dataset are shown on the left and each block represents a different step of the process, as explained in the previous sections. The images are first resized and normalized before splitting the data into the training, validation, and test sets. These are used in the hyperparameter optimization and in the training and evaluation processes. The final step, performance assessment, relies on the test set and the trained models to evaluate the accuracy and complexity of each network, as reported in the next section.

## 4 Results

### 4.1 Accuracy of the models

In this section, we compare the accuracy of the models with both datasets using data augmentation and transfer learning. Then we study the benefits of using fine-tuning. Table 3, Table 4 show the results for the Figshare and Kaggle datasets, respectively.

**Table 3:** Accuracy of the models with the Figshare dataset. The first column shows the results when the neural network is trained from scratch (SC), the second column shows the results when it is trained from scratch and using data augmentation (SC+DA), the third one corresponds to the results using transfer learning (TL), and the results in the last column use transfer learning and data augmentation (TL+DA). Bold letters highlight the best result in each column, underlined text is used for the second-best result, and italics for the third one.

Model	SC	SC+DA	TL	TL+DA
Custom	**96.7%**	95.4%	-	-
VGG16	48.0%	49.5%	**96.7%**	*94.1%*
VGG19	48.0%	49.5%	*95.4%*	92.5%
ResNet50	93.1%	41.8%	94.1%	94.5%
ResNet101	78.8%	84.6%	94.4%	**94.8%**
ResNet152	87.9%	43.3%	93.8%	92.5%
Xception	90.5%	84.0%	92.2%	90.9%
DenseNet	94.1%	**96.4%**	*95.4%*	91.9%
MobileNet	*93.5%*	93.1%	96.1%	**94.8%**
EfficientNetB0	94.1%	87.6%	96.1%	93.8%
EfficientNetB3	93.1%	*93.8%*	96.1%	92.8%
ConvNeXt	30.4%	30.1%	94.1%	89.9%

**Table 4:** Accuracy of the models with the Kaggle dataset. The first column shows the results when the network is trained from scratch (SC), the second column shows the results when it is trained from scratch and using data augmentation (SC+DA), the third one corresponds to the results using transfer learning (TL), and the results in the last column use transfer learning and data augmentation (TL+DA). Bold letters highlight the best result in each column, underlined text is used for the second-best result, and italics for the third one.

Model	SC	SC+DA	TL	TL+DA
Custom	92.7%	87.4%	-	-
VGG16	30.7%	26.9%	95.1%	89.9%
VGG19	31.6%	26.4%	*94.8%*	*92.5%*
ResNet50	*91.4%*	67.0%	92.7%	91.1%
ResNet101	84.0%	60.4%	94.2%	87.1%
ResNet152	89.6%	48.2%	93.6%	92.8%
Xception	**93.0%**	*91.1%*	92.3%	89.9%
DenseNet	91.1%	75.8%	**95.7%**	88.7%
MobileNet	90.5%	89.0%	93.9%	**93.6%**
EfficientNetB0	90.5%	**93.6%**	95.1%	92.0%
EfficientNetB3	91.1%	92.0%	94.5%	88.3%
ConvNeXt	28.4%	33.8%	93.9%	71.1%

The accuracy is in general high, with several models attaining an accuracy above 95%. The results with the Figshare dataset are slightly more accurate than with the Kaggle dataset, especially when we train from scratch. This seems reasonable because the latter has more samples, one more classification (*no tumor*), and more variability. The number of methods with an accuracy higher than 95% is seven with Figshare and three with Kaggle.

We observe that many models do not provide better results with data augmentation. This happens both when we train from scratch and when we use transfer learning. This has been pointed out in other works, like in [34], although it has not been previously shown. Probably, the reason for this behavior is that there is not an over-fitting problem and more data do not contribute to a better generalization. Note that we use dropout and a single fully connected layer, so further regularization can be detrimental. Another reason may be due to the controlled position of the patient's head in this type of image, which is similar in both the training and test sets. Rotations of the head position are unlikely to appear in the test set, thus, this transformation may not contribute to improving the variability of the training data.

The best results are typically obtained with transfer learning, except for the *Custom* model, which has not been trained on another dataset, and the *Xception* model with the Kaggle dataset, which obtained a slightly better accuracy when trained from scratch. It is noteworthy that we used weights obtained with Imagenet, which contains natural images, quite different from MR images. This denotes the generalization capability of these CNNs.

*VGG* and *ConvNeXt* are the only models with low accuracy when trained from scratch. In the case of *VGG*, this problem may be caused by the lack of skip connections that mitigate the effect of vanishing gradients. In the case of *ConvNeXt*, this may be due to fewer activation functions and normalization layers, and the substitution of BN with layer normalization.

Looking at the results of the Figshare dataset, we see that many models obtain an accuracy above 90% when trained from scratch, with *Custom*, *DenseNet*, and *EfficientNetB0* obtaining the highest accuracy of 96.7%, 94.1%, and 94.1%, respectively. The results are much better when we use transfer learning. Most of the methods obtain an accuracy above 95%, with *VGG16*, *MobileNet*, and both *EfficientNet* models obtaining an accuracy above 96%. *Xception* and *ResNet152* get the lowest accuracy.

The results with the Kaggle dataset follow a similar pattern, although the accuracy is lower than with the previous dataset in general. Similarly, the *VGG* family and *ConvNeXt* yield poor results when trained from scratch. The best results are obtained with transfer learning, especially for *DenseNet*, *EfficientNetB0*, and *VGG16*, with an accuracy of 95.7%, 95.1%, and 95.1%, respectively.

**Figure 6:**
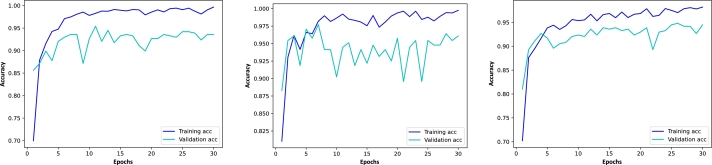
Accuracy graphics for DenseNet, EfficientNetB3, and VGG16 using the Kaggle dataset with transfer learning. The blue lines show the accuracy of the models with respect to the training set in each epoch and the cyan lines depict the accuracy with respect to the validation set.

The graphics in Fig. 6 show the evolution of accuracy during the training epochs. We observe that both the training and validation curves converge to a high accuracy and there are no overfitting problems with these models. The gap between the training and validation curves is small so we may consider that the effect of underfitting is not significant. This behavior is similar in other models. Therefore, the hyperparameters that we have chosen seem to be adequate.

These results can be improved if we use fine-tuning, as depicted in Table 5, Table 6. The first table corresponds to the Figshare dataset and the second one to the Kaggle dataset.

**Table 5:** Accuracy of the models using transfer learning and fine-tuning (Figshare dataset). The first column shows the results using transfer learning and the second column shows the results with transfer learning and fine-tuning. Bold letters highlight the best result in each column, underlined text is used for the second-best result, and italics for the third one.

Model	Without fine-tuning	With fine-tuning
Custom	**96.7%**	96.7%
VGG16	**96.7%**	97.4%
VGG19	*95.4%*	97.4%
ResNet50	94.1%	98.4%
ResNet101	94.4%	**98.7%**
ResNet152	93.8%	96.7%
Xception	92.2%	96.4%
DenseNet	*95.4%*	*98.0%*
MobileNet	96.1%	*98.0%*
EfficientNetB0	96.1%	98.4%
EfficientNetB3	96.1%	**98.7%**
ConvNeXt	94.1%	95.4%

**Table 6:** Accuracy of the models using transfer learning and fine-tuning (Kaggle dataset). The first column shows the results using transfer learning and the second column shows the results with transfer learning and fine-tuning. Bold letters highlight the best result in each column, underlined text is used for the second-best result, and italics for the third one.

Model	Without fine-tuning	With fine-tuning
Custom	92.7%	92.7%
VGG16	95.1%	96.3%
VGG19	*94.8%*	93.9%
ResNet50	92.7%	94.8%
ResNet101	94.2%	93.9%
ResNet152	93.6%	93.9%
Xception	92.3%	94.5%
DenseNet	**95.7%**	94.8%
MobileNet	93.9%	97.2%
EfficientNetB0	95.1%	*96.6%*
EfficientNetB3	94.5%	**97.5%**
ConvNeXt	93.9%	95.7%

We duplicate the results of the *Custom* model for comparison purposes only, since we did not use fine-tuning in this case. The improvement in accuracy for the rest of the models is 2.43% and 1.11% on average with the Figshare and Kaggle datasets, respectively. The major improvements are due to *ResNet50*, *ResNet101*, and *Xception* in the first case, with an increase of more than 4%, and to *MobileNet* and *EfficientNetB3*, with an increase of about 3%.

Some models provided the best improvement when we unfroze all the layers during training, such as the *ResNet*, *Xception*, and *EfficientNet* models, and others provided the best results only unfreezing the last fifteen layers. In the case of *MobileNet* and *DenseNet* the results were similar.

**Figure 7:**
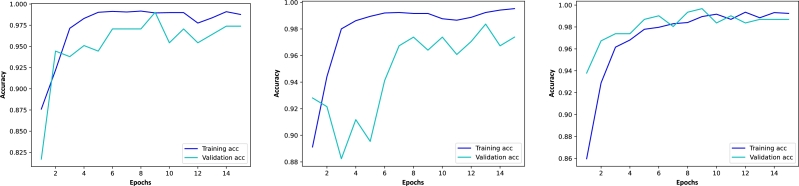
Accuracy graphics for ResNet50, MobileNet, and EfficientNetB3 using the Figshare dataset with transfer learning and fine-tuning. The blue lines show the accuracy of the models with respect to the training set in each epoch and the cyan lines depict the accuracy with respect to the validation set.

Fig. 7 shows the accuracy graphics for the ResNet50, MobileNet, and EfficientNetB3 models. In this case, the gaps between the training and validation curves are further decreased compared to the graphics in Fig. 6. The training accuracy remains high whereas the validation accuracy increases. This means that there are no over- or underfitting problems with these models, thus, we may conclude that we have chosen adequate hyperparameters. This also highlights the importance of fine-tuning the models in a second step.

**Figure 8:**
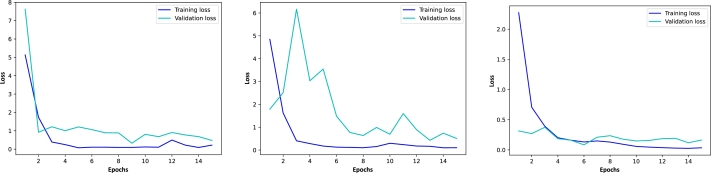
Loss graphics for ResNet50, MobileNet, and EfficientNetB3 using the Figshare dataset with transfer learning and fine-tuning. The blue lines show the loss of the models with respect to the training set in each epoch and the cyan lines depict the accuracy with respect to the validation set.

Fig. 8, on the other hand, shows the corresponding loss graphics, which confirms the convergence of the training and validation errors during the optimization process. This also verifies that there are no over- or underfitting problems.

In summary, most of the models present a high accuracy for the two datasets if we use transfer learning and fine-tuning. Data augmentation does not contribute to improving the results in general. *VGG16*, *VGG19*, and *ConvNeXt* cannot learn from scratch with these datasets, and the performance of *ResNet101* and *ResNet152* is also poor in this case. Fine-tuning is essential to obtain higher accuracy rates, providing very competitive results for several models. Various models offer outstanding performance, like *EfficientNet* and *MobileNet*, which provide high accuracy for both datasets.

**Figure 9:**
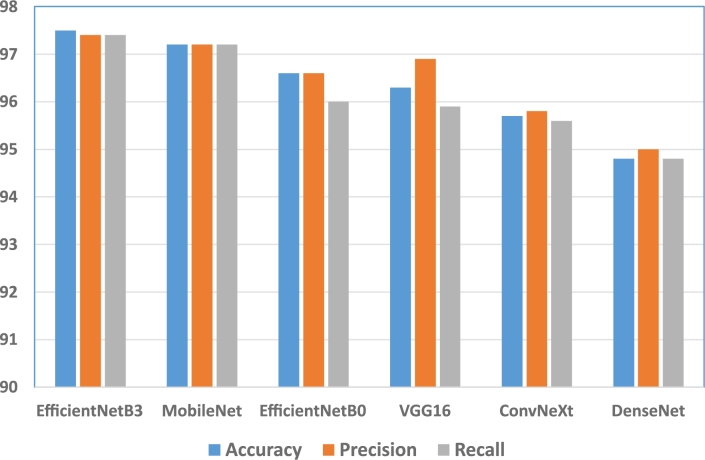
Accuracy, precision, and recall of the methods using the Kaggle dataset with transfer learning and fine-tuning.

**Figure 10:**
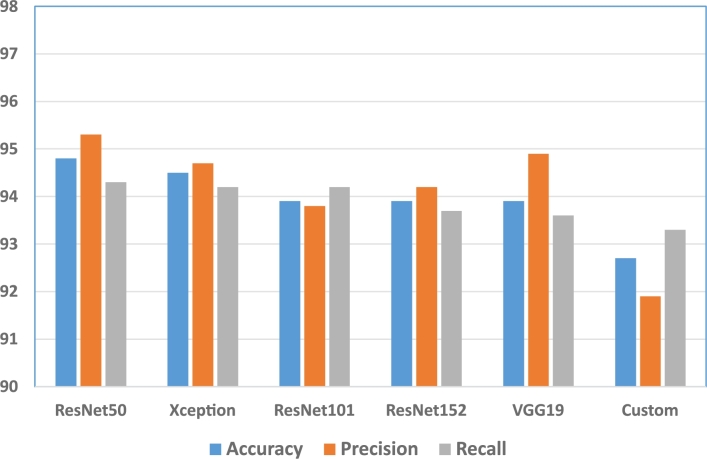
Accuracy, precision, and recall of the methods using the Kaggle dataset with transfer learning and fine-tuning. The metrics of the *Custom* model correspond to the result when training from scratch.

Figure 9, Figure 10 show the accuracy, precision, and recall of the methods. In this case, we show the results for the Kaggle dataset corresponding to the transfer learning and fine-tuning setting, except for the *Custom* model for which we have taken the results when trained from scratch.

The best models are *EfficientNetB3* and *MobileNet*, with the highest precision and recall, followed by *EfficientNetB0* and *VGG16*. The precision and recall of the models are similar, except for the *VGG16*, *ResNet50*, and *VGG19* models, where the precision is significantly bigger than their recall. This means that these models have a tendency to classify fewer samples as false positives, which indicates that they are more confident about the results.

On the other hand, the recall of the *Custom* model is higher than its precision, which means that the rate of false negatives is smaller in this case, so it tends to detect a larger rate of true positive cases.

### 4.2 Analysis of tumor classification

Table 7 shows the precision of the models concerning each type of tumor. The *pituitary* tumor class obtains the highest rates, with two models attaining an accuracy of 100%. This label has an average precision of 97.9%, with *ResNet152* and *ResNet50* providing the lowest precision of 95.4% and 96.5%, respectively. This result is reasonable because the location of this type of tumor is limited to the pituitary gland. The second-best label is *no tumor* with a precision of 95.3%, followed by *gliomas* with 94.3%, and *meningiomas* with 93.8%.

**Table 7:** Tumor type precision with the Kaggle dataset. This table shows the precision of the models concerning each type of tumor. The *meningioma* class is the label with the lowest precision in general, whereas the *pituitary* tumor class is the label with the highest precision. Bold letters highlight the best result in each column, underlined text is used for the second-best result, and italics for the third one.

Model	Glioma	Meningioma	No tumor	Pituitary
Custom	93.3%	92.2%	84.5%	*97.8%*
VGG16	**97.8%**	91.5%	98.0%	**100.0%**
VGG19	88.9%	94.3%	**100.0%**	96.6%
ResNet50	91.5%	95.9%	*97.3%*	96.5%
ResNet101	96.8%	88.9%	91.8%	97.6%
ResNet152	94.2%	91.3%	96.0%	95.4%
Xception	93.9%	91.8%	95.6%	97.6%
DenseNet	95.1%	91.3%	95.7%	*97.8%*
MobileNet	94.7%	**97.9%**	96.2%	**100.0%**
EfficientNetB0	95.7%	*96.0%*	95.9%	98.8%
EfficientNetB3	*96.7%*	**97.9%**	96.2%	98.8%
ConvNeXt	92.7%	96.8%	96.1%	97.7%

Average	94.3%	93.8%	95.3%	97.9%
Std. Deviation	2.38%	2.89%	3.72%	1.31%

The samples of the Kaggle dataset are distributed as follows: *gliomas* with 28.4%, *meningiomas* with 28.7%, *no tumor* with 15.3%, and *pituitary* tumors with 27.6%. In this case, we observe that the *no tumor* class has fewer samples than the other labels, which may affect the performance of the models. Additionally, many images with this label look brighter than the images of other classes; see Fig. 2.

There are various models, such as *EfficientNetB0* and *EfficientNetB3*, that provide similar precisions for all the labels, whereas other models, such as *VGG16* and *VGG19*, provide higher precisions for *no tumor* and *pituitary* tumors.

The most difficult tumors to classify are *meningiomas*, with only four models—*MobileNet*, *EfficentNetB3*, *ConvNeXt*, and *EfficentNetB0*—obtaining an accuracy higher than 96%.

**Figure 11:**
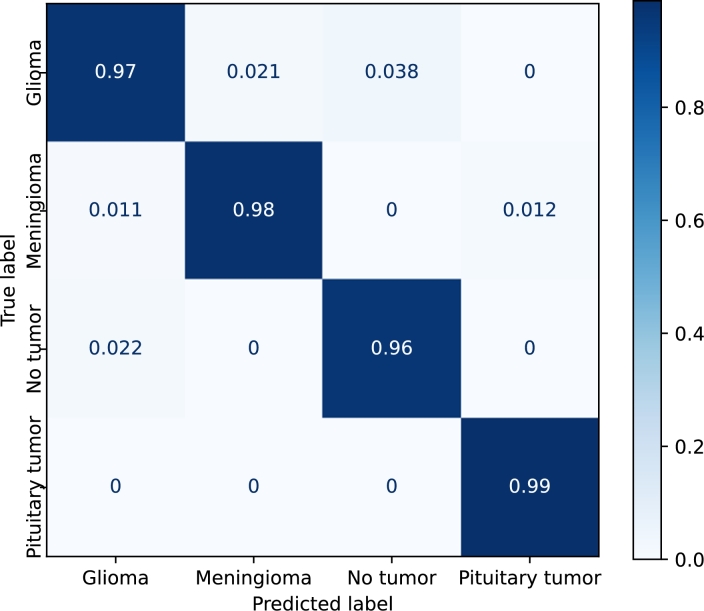
Confusion matrix corresponding to the *EfficientNetB3* model using the Kaggle dataset with transfer learning and fine-tuning.

**Figure 12:**
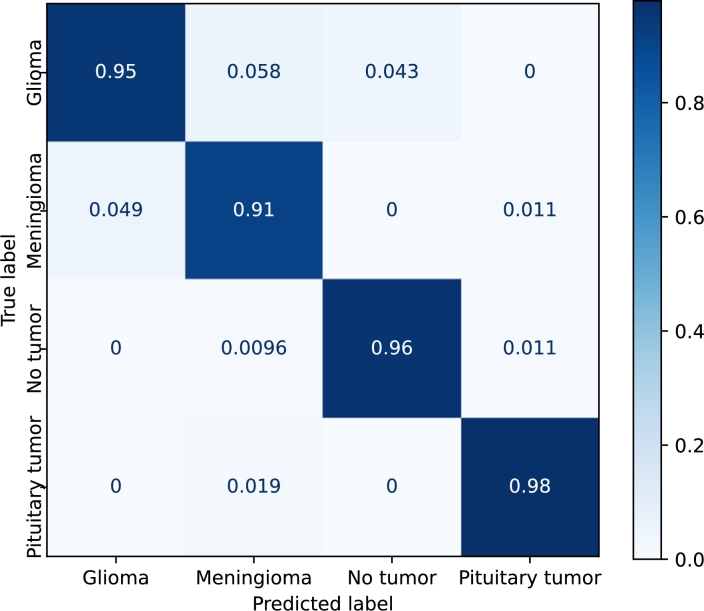
Confusion matrix corresponding to the *DenseNet* model using the Kaggle dataset with transfer learning and fine-tuning.

**Figure 13:**
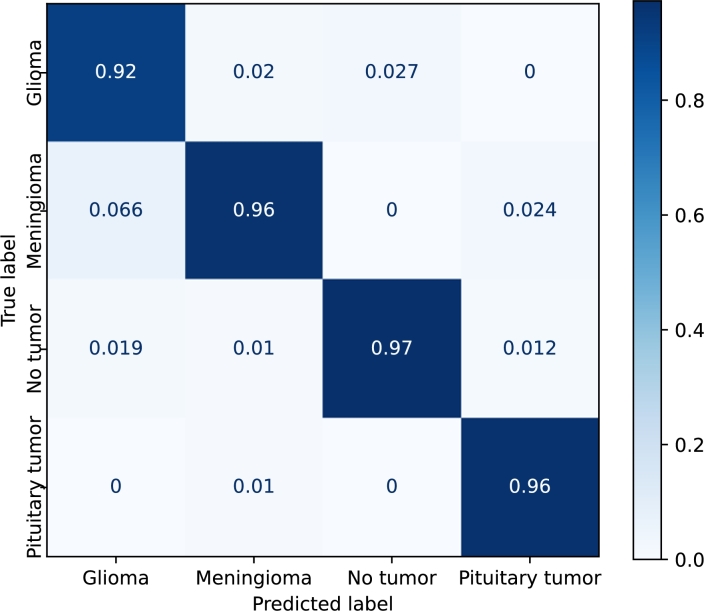
Confusion matrix corresponding to the *ResNet50* model using the Kaggle dataset with transfer learning and fine-tuning.

**Figure 14:**
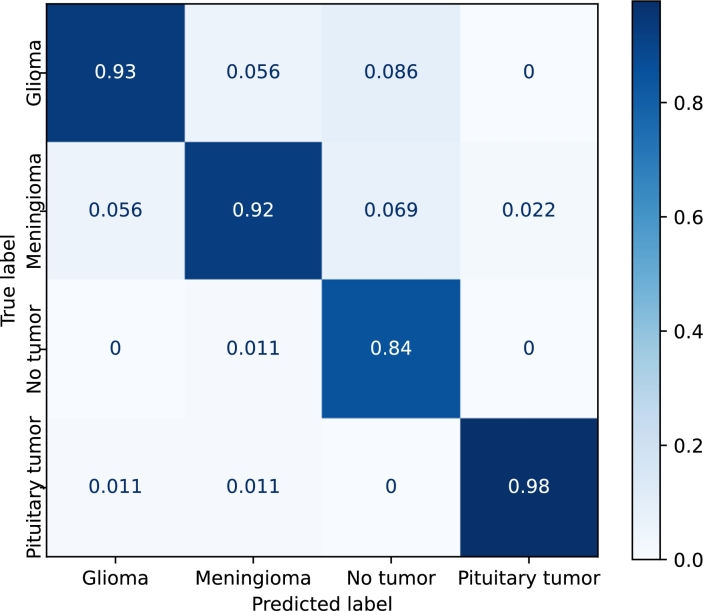
Confusion matrix corresponding to the *Custom* model using the Kaggle dataset.

Figure 11, Figure 12, Figure 13, Figure 14 show the confusion matrices of the *EfficientNetB3*, *DenseNet*, *Resnet50*, and *Custom* models, respectively. *EfficientNetB3* and *Custom* provide the best and worst precisions, respectively, and the results of *DenseNet* and *ResNet50* are somewhere in the middle.

The prediction of *pituitary* tumors is robust for all the models. *Meningiomas*, and to a lesser extent *no-tumors*, are sometimes classified as pituitary tumors. *Gliomas*, on the other hand, are never confused with *pituitary* tumors. The precision of the *no-tumor* label is also high except for the *Custom* model. The false positives, in this case, are mainly due to *gliomas*. *Glioma* is the class that produces the largest number of false positives, and *meningiomas* are mainly confused with *gliomas* in all the models.

*EfficientNetB3* provides similar results for all the labels. The main misclassifications are produced by *gliomas*, which is confused with *meningiomas* and *no-tumors*. In the case of *DenseNet* and *ResNet50*, the major confusion is between *gliomas* and *meningiomas*.

### 4.3 Analysis of model complexity

Table 8 shows the complexity of the models in terms of size and running times. It details the accuracy obtained with the Kaggle dataset, obtained from Table 6, the number of parameters, and the training and inference times in each case. We also show the throughput, which is the inverse of the inference time and represents the number of images that can be processed by the model per second. This information is somehow more intuitive than the inference time since a larger value means better performance.

**Table 8:** Complexity of the models. The first column shows the accuracy of the models with the Kaggle dataset using transfer learning and fine-tuning, the second column depicts the number of parameters of each model, the third column stands for the training times expressed in seconds per epoch, the fourth column details the time necessary to process one image in inference mode, and the last column represents the image throughput. Bold letters are used to highlight the best result in each column, underlined text for the second-best result, and italics for the third one.

Model	Accuracy	**Table:** #parameters (Millions)	**Table:** Training time (seconds/epoch)	**Table:** Inference time (milliseconds/image)	**Table:** Throughput (images/second)
Custom	92.7%	*10.7M*	29.6 s	**4.3 ms**	**234.37**
VGG16	96.3%	165.7M	31.0 s	5.8 ms	171.02
VGG19	93.9%	171.0M	36.3 s	6.8 ms	147.16
ResNet50	94.8%	23.6M	*26.7 s*	4.7 ms	210.93
ResNet101	93.9%	42.6M	36.6 s	6.8 ms	147.16
ResNet152	93.9%	58.3M	51.6 s	9.3 ms	107.25
Xception	94.5%	20.9M	33.4 s	6.0 ms	166.53
DenseNet	94.8%	18.3M	36.1 s	6.6 ms	150.67
MobileNet	97.2%	**3.2M**	**23.7 s**	4.4 ms	226.00
EfficientNetB0	*96.6%*	5.9M	25.4 s	*4.5 ms*	*218.21*
EfficientNetB3	**97.5%**	12.9M	27.0 s	5.2 ms	191.76
ConvNeXt	95.7%	27.8M	58.2 s	10.3 ms	97.35

The best-performing methods must present a high accuracy and a large throughput, additionally with a small training time. The first two metrics allow us to choose the best model for production, whereas the third one complements this information to understand the cost of updating the parameters of the models in the future.

Looking at the table, we observe a large variability in the size of the networks. The smallest ones are *MobileNet* and *EfficientNetB0* with 3.2M and 5.9M parameters, and the biggest ones are *VGG19* and *VGG16* with 171M and 165.7M parameters, respectively.

Training times are calculated as the average run-time of 10 epochs. These times are calculated with transfer learning, except for the *Custom* model that we used in the SC configuration. The lowest training times are given by *MobileNet*, *EfficientNetB0*, and *ResNet50*. The highest ones are given by *ConvNeXt* and *ResNet152*.

The size of the networks does not necessarily correlate with training times. This is the case, for instance, of the *VGG* models, which are comparable with smaller networks, such as *DenseNet* or *Xception*. In this regard, *VGG* has the best trade-off between training time and number of parameters, whereas the worst is given by *ConvNeXt* and *DenseNet*, which have a small size in comparison with their training times. *EfficientNetB3*, on the other hand, is comparatively worse than *ResNet50* and *Xception* in this respect.

The average inference times and throughput are calculated using all the images of the dataset. The best throughputs are typically associated with networks with a small number of parameters, with the *Custom* and *MobileNet* models having the best rates, followed by *EfficientNetB0*. The *Custom* network provides the best rate even with many more parameters than *MobileNet*, which is indicative of its simplicity. The *VGG* architecture has a significant throughput compared to its size. ConvNeXt provides the worst throughput.

**Figure 15:**
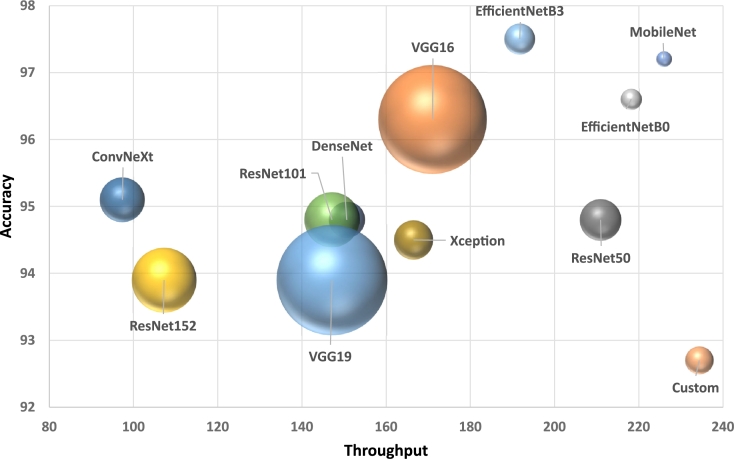
Accuracy, throughput, and number of parameters of the neural networks. The size of each bubble represents the number of parameters of each model. The best models are situated on the top-right position of the graphic, with high accuracy and throughput.

Fig. 15 shows the relation between the accuracy, throughput, and size of the methods. This is illustrative of the overall performance of the models: the best models for this problem should have high accuracy and large throughput with a relatively small size. This is the case, for instance, of the *MobileNet*, *EfficientNetB3*, and *EfficientNetB0* models. *VGG16* has the second largest size, although its throughput is in the middle of the ranking and its accuracy is in the top four.

We observe that the best-performing networks have a small number of parameters, especially *MobileNet*, with only 3.2M. Thus, we may conclude that these datasets do not require models with large capacity.

The worst-performing methods are situated in the bottom-left corner of the graphic, such as *ResNet152*, *VGG19*, or *ConvNeXt*. *ResNet101* and *DenseNet* are slightly more accurate than *VGG19* but are comparable in terms of throughput.

### 4.4 Comparison with state-of-the-art

Table 9 compares our results with SOTA methods. The table is divided into two blocks, with the upper part showing the SOTA results and the lower part showing our results extracted from Table 3, Table 4.

**Table 9:** Comparison with SOTA methods. At the top, we show the SOTA results and, at the bottom, our results from Table 3, Table 4. The second column summarizes the techniques used in each method, the third column shows the image size used in each method when it is available, and the fourth and fifth columns show the accuracy with the Figshare and Kaggle datasets, respectively. Bold letters highlight the best result in each column, underlined text is used for the second-best result, and italics for the third one.

Method	Techniques	Image Size	Figshare Acc.	Kaggle Acc.
Tahir, et al. (2019) [7]	2D DWT, PCA, SVM	512 × 512	86.0%	-
Afshar, et al. (2018) [31]	Capsule Network	128 × 128	86.6%	-
Afshar, et al. (2019) [32]	Capsule Network, Segmentation	128 × 128	90.9%	-
Cheng, et al. (2015) [9]	Intensity Histogram, GLCM, BoW	512 × 512	91.3%	-
Ismael et al. (2018) [22]	2D DWT, 2D Gabor filter, MLP	-	91.9%	-
Zhou, et al. (2018) [41]	DenseNet and LSTM	-	92.1%	-
Pashaei, et al. (2018) [17]	Convolut., BN, MaxPool, ELM	28 × 28	93.7%	-
Ayadi, et al. (2021) [26]	Convolutional, BN, MaxPool	256 × 256	94.7%	-
Phaye, et al. (2018) [33]	Dense Capsule Network	-	95.0%	-
Ghassemi, et al. (2020) [40]	Generative Adversarial Network	64 × 64	95.6%	-
Shaik et al. (2022) [42]	Xception, Attention, ConvLSTM	-	96.5%	-
Badža et al. (2020) [25]	Convolutional, Dropout, MaxPool	256 × 256	96.6%	-
Kumar, et al. (2021) [34]	ResNet50	-	97.1%	-
Amin, et al. (2020) [67]	GoogLeNet, KNN	224 × 224	98.0%	-
Bodapati, et al. (2021) [43]	Xception and InceptionResNetV2	-	98.0%	-
Tummala, et al. (2022) [39]	Vision Transformer	384 × 384	98.2%	-
Mehnatkesh et al. (2023) [36]	ResNet50, Ant Colony Optim.	224 × 224	98.7%	-
Polat et al. (2021) [5]	ResNet50	224 × 224	**99.0%**	-
Hossain, et al. (2023) [45]	VGG16, Inception, Xception, ViT	224 × 224	-	96.5%
Goutham, et al. (2022) [35]	EfficientNetB0	150 × 150	-	96.9%
Saleh, et al. (2020) [6]	Xception	256 × 256	-	**98.8%**
Mishra, et al. (2022) [68]	EfficientNetB2	150 × 150	-	**98.8%**

*VGG16*	3 × 3 kernels, 16 layers	256 × 256	97.4%	96.3%
*DenseNet*	Dense skip connections, bottleneck layers, 201 layers	256 × 256	98.0%	94.8%
*MobileNet*	bottleneck depth-separable convolutions with residuals,	256 × 256	98.0%	*97.2%*
*ResNet50*	Skip connections, pre-activation residual functions, 50 layers	256 × 256	*98.4%*	94.8%
*EfficientNetB0*	Inverted bottlenecks and squeeze-and-excitation optimization	256 × 256	*98.4%*	96.6%
*ResNet101*	Skip connections, pre-activation residual functions, 101 layers	256 × 256	98.7%	93.9%
*EfficientNetB3*	Inverted bottlenecks and squeeze-and-excitation optimization	256 × 256	98.7%	97.5%

For each method, we summarize the techniques used in the corresponding article, the size of the input image, and the accuracy obtained with the Figshare and Kaggle datasets. This latter dataset was released in 2020, thus the number of works is smaller. We do not report the size of the images of various works because it is not documented in the corresponding article.

The best methods in the literature for Figshare are based on *ResNet50*, Vision Transformers, and *Xception*. Nevertheless, we observe that our results for *EfficientNetB3*, *ResNet101*, *EfficientNetB0*, and *ResNet50* are in the top of the ranking. Our experiments confirm the performance of *EfficientNet* and *ResNet50*.

The first method [5], with an accuracy of 99%, obtained the best result with *ReNet50* using transfer learning. They included three fully connected layers on top of the network and used the Adadelta optimizer. In our study, we also tested a similar configuration but did not obtain such results. On the contrary, the training produced overfitting problems that were solved losing some precision. The authors did not explain how they addressed these problems. Another drawback of this work is the large number of parameters, with 98M.

The second-best result on Figshare [36] also relies on *ResNet50*. The authors found optimal hyperparameters for the neural network using a metaheuristic method based on the ant colony optimization algorithm. We did not search for optimal configurations in this study, so it is probable that we may obtain slightly better results with other hyperparameters.

In the case of Kaggle, our result with *EfficientNetB3* is close to the first method and our result with *MobileNet* is also competitive. The top methods [68], [6] rely on *EfficientNetB2* and *Xception* using transfer learning, with an accuracy of 98.8%. The first one combines two different datasets, creating a large training set with many more samples than in the Kaggle dataset. Therefore, it is difficult to compare with this work. Nevertheless, they used an EfficientNet model, which is in line with our results. On the other hand, the authors do not detail the configuration of the network or the training parameters, so it is difficult to reproduce the results. The second work also has the same drawbacks, with many images in the training set not only corresponding to the original Kaggle dataset. It does not provide enough details to reproduce the results either.

## 5 Discussion

This work demonstrates that several standard CNNs can provide high accuracy with brain tumor datasets. This contrasts with many previous methods that propose complex architectures or pipelines that cannot usually be trained end-to-end. These neural networks extract adequate features from the input images without any pre-processing technique.

The best-performing networks were *EfficientNet*, and *MobileNet*, ranking in the top of the SOTA. *ResNet* and *DenseNet* performed very well with the Figshare dataset but they obtained poor results with the Kaggle dataset. This is probably due to the larger variability of images and the additional *no-tumor* class.

The *Custom* network provided one of the best results with Figshare, but it performed poorly with Kaggle. When we used fine-tuning, this network remained in the last positions in both cases. The best networks in the *ResNet* family were *ResNet101* and *ResNet50*, respectively, obtaining a significant improvement using fine-tuning with Figshare. *VGG16* was the clear winner in the VGG architecture and *DenseNet* ranked in the middle of the classification with *Xception* and *ResNet50*. Although *ConvNeXt* is a recent neural network, it does not perform satisfactorily in this problem.

Many networks yielded good results when trained from scratch; however, *VGG* and *ConvNeXt* were the only two networks that obtained poor results in this case.

Transfer learning was key to improving the accuracy of the models, especially for *VGG* and *ConvNeXt*. Most models improved by a significant percentage in this case. Additionally, fine-tuning allowed the models to improve further, obtaining state-of-the-art results in various cases. Some models provided the best results by unfreezing all the layers, whereas others hardly improved by unfreezing the last layers, like *VGG* and *ConvNeXt*. The *Xception* network was one of the models that obtained the best improvement with fine-tuning, but it did not rank at the top of the classification.

Our experiments showed that data augmentation did not contribute to improving the results. This probably means that there was not an overfitting problem with these datasets, or that dropout was sufficient to overcome this problem. This is reasonable as we only used one fully connected layer on top of the models.

The analysis concerning each type of tumor revealed that *pituitary* tumor is the easiest label to classify and *meningioma* is the most difficult one. The two labels that are most often confused are *meningiomas* and *gliomas*. *Gliomas* are also confused to a lesser extent with the *no-tumor* class.

The complexity analysis was important to understand other factors involved in network performance. We showed that the best performance corresponds to smaller networks, like *MobileNet* or *EfficientNetB0*, which yielded very competitive results in both datasets. We note that most of the parameters in these models came from the fully connected layers on top of the networks, which are usually the ones that cause overfitting.

We only used one fully connected layer, whereas other works in the literature typically used a few more. This allowed us to reduce the size of the networks although still providing competitive results. We saw, in the comparison with SOTA methods, that the most accurate ones had many more parameters for slightly better precision. Furthermore, the methods that ranked first were also trained with many more images from multiple datasets, and, in some cases, the configuration of the hyperparameters was not clear.

Our results with the Figshare dataset were consistently better than with Kaggle, which is probably due to the variability of the images in the latter. There is an important overlap between the two datasets but there are many images from other sources in the Kaggle dataset and an additional classification.

### 5.1 Limitations

It is not possible to test all configurations for all the models and find the best results in each case. Hence, this work has to be seen as a thorough study to rank the models using homogeneous conditions. Nevertheless, the best-performing methods are excellent candidates for usage in a real scenario.

The results are promising but are linked to the databases we have used. The kind of information in the Figshare dataset is quite homogeneous, so we may not expect that the models trained with it will maintain their performance with other types of MR images. In the case of the Kaggle dataset, since there is more variability, we may expect better behavior in this regard.

According to the World Health Organization (WHO), there are many more types of brain tumors, but current datasets only contain a few classes. We cannot ensure that the performance of the models will remain the same using more labels, although it is reasonable to think that this should affect all the models similarly. It is an important issue for the future to create datasets with images from different MRI scanners and more types of classifications.

Another limitation, especially when comparing with the state of the art, is the lack of a test set for which the labels are unknown. It would be interesting to rely on an external evaluation system to assess the performance of the methods so that the comparison can be made under the same conditions.

The accuracy of the models can be further improved with other techniques, such as *early stopping*. During the training process, some models provided better results before the end of the last epoch, so the result with the test set would probably have provided better results in some cases.

Another conclusion from our study is that data augmentation seems not to improve the results in general, but we have tested three types of transformations: rotations, image scaling, and horizontal flipping. We cannot ensure that other data augmentation techniques, such as brightness or contrast changes, or even smaller amounts of transformations, may yield better results in this case.

## 6 Conclusion

In this work, we assessed the performance of the most relevant convolutional neural networks for the classification of brain tumors and showed that several networks provided good accuracy.

We used several techniques for training the models, like transfer learning, data augmentation, and fine-tuning. Although some models provided good results when trained from scratch, most of the models obtained better performance with transfer learning. Fine-tuning was also important to get an additional improvement of up to 4% in some cases, although a few networks did not get any improvement. Data augmentation, on the other hand, did not contribute to increasing the accuracy of the models in general.

Our study also evaluated the performance of the models in terms of image throughput and network capacity. We showed that various networks with a reduced number of parameters and large throughput, like *EfficientNetB3* or *MobileNet*, offered outstanding results.

The performance of the models is high and we should expect a similar behavior for larger datasets. However, in future works, we will study the ability of these networks to deal with more labels and images. We will use multiple brain tumor datasets and extend the study to more neural networks. In particular, we are interested in analyzing the performance of vision transformers, for which we have not obtained satisfactory results yet. We are also interested in applying self-supervised learning techniques and generative models for the classification of brain tumors.

## Additional information

No additional information is available for this paper.

## Declaration of generative AI and AI-assisted technologies in the writing process

During the preparation of this work the authors used Grammarly, Bing Chat, and ChatGPT in order to improve language and readability. After using these tools, the authors reviewed and edited the content as needed and take full responsibility for the content of the publication.

## CRediT authorship contribution statement

**Daniel Reyes:** Conceptualization, Data curation, Formal analysis, Investigation, Methodology, Software, Validation, Writing – original draft. **Javier Sánchez:** Conceptualization, Data curation, Formal analysis, Funding acquisition, Investigation, Methodology, Project administration, Software, Supervision, Validation, Writing – original draft, Writing – review & editing.

## Declaration of Competing Interest

The authors declare the following financial interests/personal relationships which may be considered as potential competing interests: Javier reports financial support was provided by Canary Islands Health Service.

## Data Availability

Data included in article/supp.material/referenced in article.

## Footnotes

1 https://www.kaggle.com/datasets/sartajbhuvaji/brain-tumor-classification-mri.

2 https://github.com/jsanchezperez/brain_tumor_classification.
